# Childhood and Adulthood Severe Stressful Experiences and Biomarkers Related to Glucose Metabolism: A Possible Association?

**DOI:** 10.3389/fpsyt.2021.629137

**Published:** 2021-05-14

**Authors:** Sarah Tosato, Chiara Bonetto, Nicola Lopizzo, Nadia Cattane, Mara Barcella, Giorgia Turco, Mirella Ruggeri, Stefania Provasi, Simona Tomassi, Paola Dazzan, Annamaria Cattaneo

**Affiliations:** ^1^Section of Psychiatry, Department of Neuroscience, Biomedicine and Movement Sciences, University of Verona, Verona, Italy; ^2^Biological Psychiatry Unit, IRCCS Istituto Centro San Giovanni di Dio Fatebenefratelli, Brescia, Italy; ^3^Department of Pharmacological and Biomolecular Sciences, University of Milan, Milan, Italy; ^4^Department of Psychological Medicine, Institute of Psychiatry, Psychology and Neuroscience, King's College, London, United Kingdom

**Keywords:** childhood trauma, glucose, health outcome, insulin, stressful life events

## Abstract

**Background:** No study investigated the association between stress exposure in different stages of life and metabolic dysfunction.

**Aim:** We explore the association between stress exposure and several biomarkers related to glucose metabolism (insulin, c-peptide, GIP, GLP-1, glucagon) in a group of 72 healthy individuals.

**Method:** We used the Childhood Experience of Care and Abuse-Questionnaire (CECA-Q) and a modified version of the Life Events Scale to define exposure to stress, according to four categories: no exposure to childhood trauma (CT) nor to stressful life events (SLEs) (46%), only to CT (25%), only to SLEs (21%), to both (8%).

**Results:** We found that c-peptide (*p* = 0.006) and insulin (*p* = 0.002) levels differed among the four categories: 0.77 ng/ml (SD 0.27) and 0.21 ng/ml (SD 0.06) for none, 0.77 (SD 0.37) and 0.20 (SD 0.08) for only SLEs, 0.88 (SD 0.39) and 0.27 (SD 0.12) for only CT, 1.33 (SD 0.57) and 0.40 (SD 0.28) for both, respectively. The highest levels of biomarkers were found in subjects exposed to both CT and SLEs.

**Conclusion:** Our preliminary results seem to suggest that CT might be specifically associated with a dysfunction of glucose metabolism, which might increase the risk of poorer health outcomes in adulthood. This association seems to be even stronger in individuals additionally exposed to SLEs in adulthood. In conclusion, if confirmed in other studies, subjects exposed to both CT and SLEs appear the most vulnerable individuals, for whom preventative interventions, such as healthy lifestyle education programs, might ameliorate the risk of developing metabolic abnormalities.

## Introduction

Childhood trauma (CT) can increase vulnerability to several medical conditions in adulthood, such as cardiovascular diseases, type 2 diabetes, and metabolic disorders (1). More specifically, victims of childhood sexual and physical abuse have been found to be more likely to be obese or to show symptoms of metabolic syndrome (2), and a history of maltreatment has been associated with higher levels of metabolic markers in adulthood, including glycated hemoglobin (3). Evidence also suggests that CT increases the lifetime risk of developing type 2 diabetes by up to 32% (4).

A possible biological mechanism through which CT may increase the risk of metabolic disorders in adulthood is via an effect on the glucose metabolic pathway (5). CT may act as a severe and chronic stress that induces the production of elevated circulating glucose and insulin (5). Insulin resistance can contribute to visceral obesity or result from obesity itself, a key feature of the metabolic syndrome (6). Moreover, the hypothalamic-pituitary-adrenal (HPA) axis and the immune response, both activated by CT, can interact with other stress markers and hormones involved in glucose metabolism, like insulin, glucagon, secretin, gastric inhibitory polypeptide (GIP), and glucagon-like peptide-1 (GLP-1) (7), which further regulate HPA axis homeostasis. Insulin is known to exert an inhibitory action on HPA axis tone (8), while glucagon, GLP-1 and GIP may enhance its function inducing the release of corticotrophin release hormone (CRH)/adrenocorticotrophic hormone (ACTH) (7). GIP and GLP-1 are hormones which are released by the gastrointestinal system in response to the glucose intake and in turn stimulate the release of insulin, causing the so called “incretin effect” (9). To date, only one study has investigated the relationship between CT and some biomarkers related to glucose metabolism, and has reported no association between insulin and physical and sexual abuse exposures (10).

Stressful life events (SLEs) in adulthood have also been found to increase the risk for metabolic syndrome and type 2 diabetes (11, 12). As for CT, the biological response to SLEs mainly involves the HPA axis, inducing the release of cortisol, which in-turn may be responsible for central adiposity and insulin resistance (13). Interestingly, a recent study has found that the experience of SLEs in the previous 12 months is associated with an increased risk of type 2 diabetes, and suggested that chronic exposure to SLEs could lead to a progressive increase in insulin resistance, dysglycemia, glucotoxicity, beta cell dysfunction, and later type 2 diabetes (14).

However, to the best of our knowledge, no study has investigated whether exposure to severe stress in two different stages of life (childhood or/and adulthood) is associated with a metabolic dysfunction in adulthood, and whether exposure to both significantly increases this association. In this study we explored, in a sample of healthy individuals, if exposure to CT would be associated with higher levels of biomarkers of glucose metabolism that regulate HPA axis homeostasis, specifically insulin, c-peptide, GIP, GLP-1, glucagon. Moreover, we explored and if this association would be more evident in those individuals who additionally experienced severe SLEs in adulthood.

## Methods

### Participants and Clinical Assessments

We recruited 72 subjects through advertisement posted at the University Hospital of Verona (Italy). They had a mean age of 37 years (SD 12), 46% were males, and they had a mean BMI of 24.3 (SD 4.3). They were all negative for physical or psychiatric disorders, traumatic brain injury, intellectual disability, pregnancy, breastfeeding and were not taking any medication. The absence of psychiatric disorders was ascertained using the Mini International Neuropsychiatric Interview (M.I.N.I. Plus) (15), the Mini International Neuropsychiatric Interview (SCID-II) (16), the Hamilton Rating Scale for Depression (HAM-D) (17) and the Bech-Rafaelsen Mania Rating Scale (BRMRS) (18). The study was approved by the Ethic Committee of the Verona University Hospital (N. 1995, 31/08/2011; N. 1816, 29/05/2013) and all participants gave written informed consent.

CT was derived from the Childhood Experience of Care and Abuse-Questionnaire (CECA-Q) (19), which is a semi-structured interview used to assess several adversities including emotional (antipathy and neglect) abuse, loss and separation, and physical and sexual abuse that occurred before age 17. For the aim of the present study, was defined CT as having experienced at least one among physical abuse (repeated exposure to physical violence from parental figures), sexual abuse (unwanted sexual experiences) and loss (having experienced death of one or both parents) or separation (a detachment from at least one of the parental figures longer than 6 months).

Severe SLEs in the previous 6 months were evaluated by a modified version of the Life Events Scale (20), and included death of a family member, sexual or physical abuse, being accused of having committed a crime, sentence of imprisonment, being exposed to war or natural catastrophes, family breakdown, being removed from home, sentimental breakdown, severe physical illness (21, 22). We defined four categories of stress exposure: none, only CT, only SLEs, or both.

### Metabolic Biomarkers

Blood samples were collected in the morning, while fasting. Tubes were kept at room temperature for 2 h, followed by 1 h at 4°C, then centrifuged for plasma separation (3,000 g for 15 min). The concentration of metabolic biomarkers were determined using Bio-PlexPro™ Human Diabetes Assays on a Bio-Plex 200 System array reader (Bio-Rad, CA, USA). Briefly, 12.5 μl of each sample were diluted 1:4 with the sample diluents, and then 50 μl of the diluted sample were incubated in a pre-wet filter plate for 1 h in the dark with the biotinylated detection antibody. Each analyte was detected by the addition of a streptavidin-phycoerythrin solution and quantified using the BioPlex array reader (Bio-Rad, CA, USA). Data acquisition and analysis from the reactions were performed using a Bio-Plex system reader. Standard curves were obtained using as reference the model given by the manufacturer. The different analytes concentrations (ng/ml) were calculated using the Bio-Plex Manager software. All measurements were performed in duplicates and all the samples with different clinical features and negative control samples were run together in the same plates.

### Statistical Analysis

Biomarker plasma level outliers (Q1-1.5^*^IQR, Q3+1.5^*^IQR) were removed. Comparisons among the four exposure groups were performed by ANOVA and Chi-square, where appropriate. All *p*-values were two-tailed with a significance level of 0.05. Due to the exploratory nature of the study, we did not apply a correction for multiple comparisons. Analyses were performed by using SPSS 22.0.

## Results

**Figure 1:**
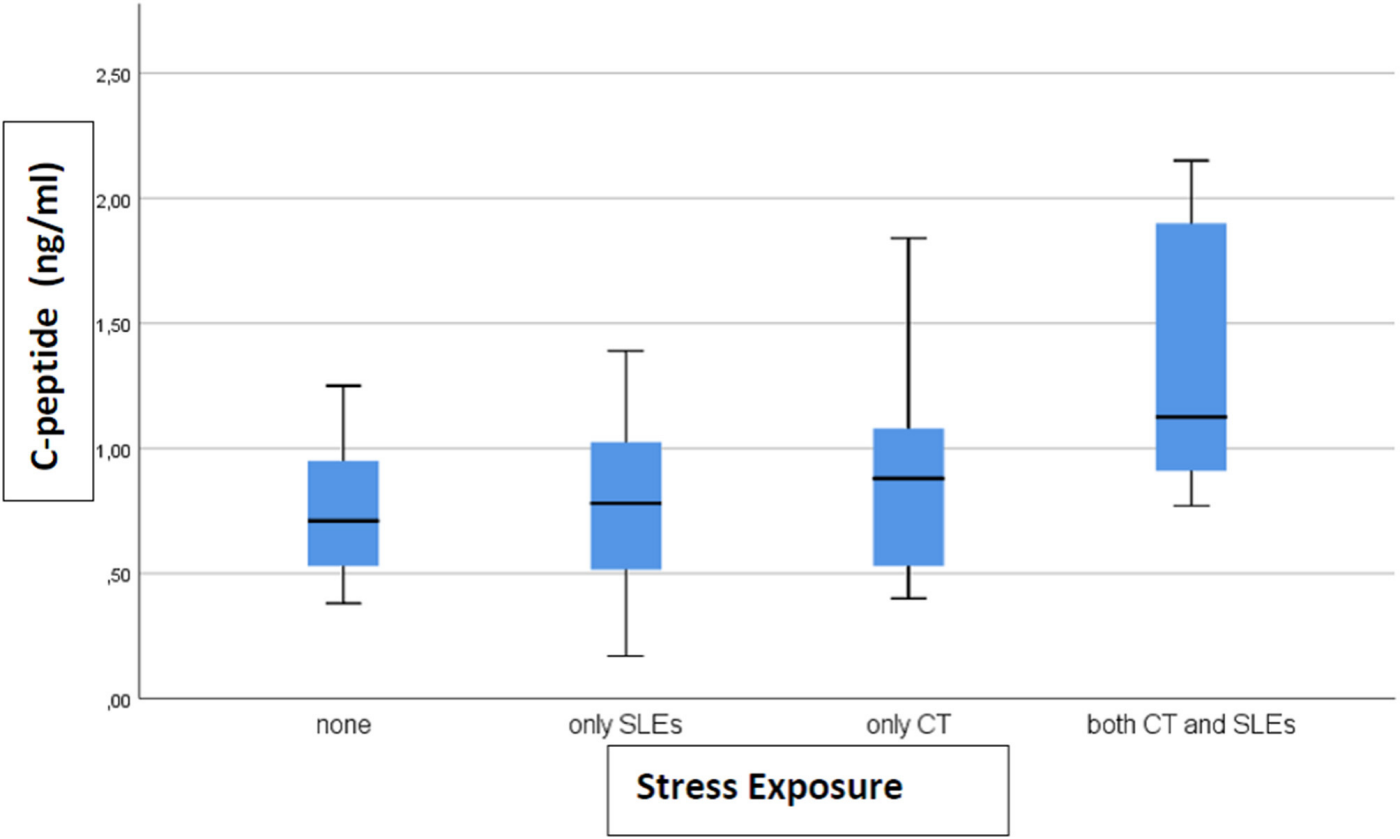
The box-plot for c-peptide serum concentration distribution in subjects who experienced different exposure to childhood trauma (CT) and severe stressful life events (SLEs) (*n* = 72).

**Figure 2:**
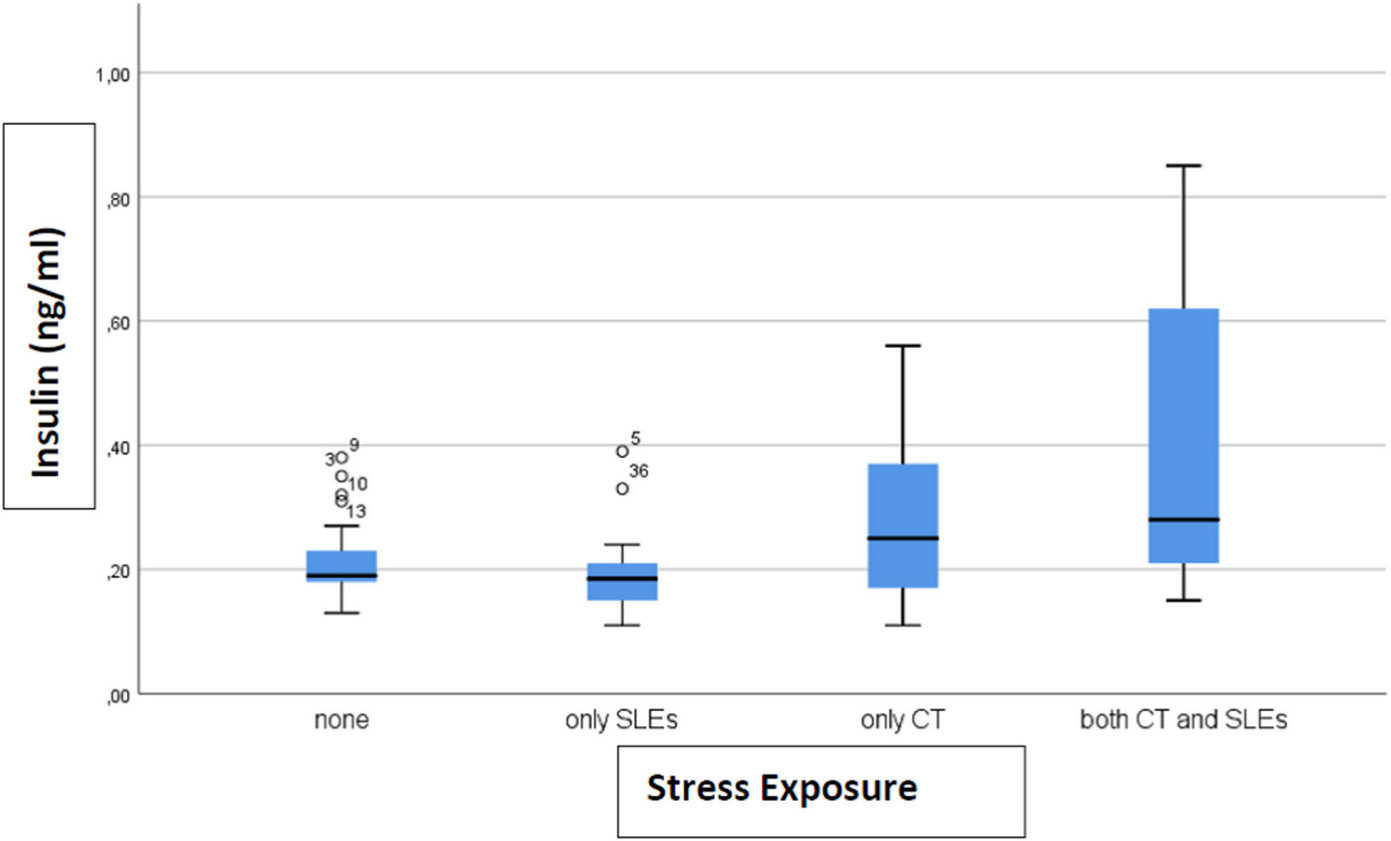
The box-plot for insulin serum concentration distribution in subjects who experienced different exposure to childhood trauma (CT) and severe stressful life events (SLEs) (*n* = 72).

A total of 33 (46%) subjects reported no stress exposure, 18 (25%) only to CT, 15 (21%) only to SLEs and 6 (8%) to both, with no difference in age, sex, and BMI between the groups (data not shown). Only levels of c-peptide (*p* = 0.006) and insulin (*p* = 0.002) differed between groups. The level of c-peptide (ng/ml) (after removing 1 outlier) was 0.77 (SD 0.27) in the not exposed group, 0.77 (SD 0.37) in SLEs only, 0.88 (SD 0.39) in CT only, and 1.33 (SD 0.57) in both CT and SLEs (Figure 1). For insulin, the levels (ng/ml) (after removing 4 outliers) were 0.21 (SD 0.06) for none, 0.20 (SD 0.08) for SLEs only, 0.27 (SD 0.12) for CT only, and 0.40 (SD 0.28) for both (Figure 2). Our results indicate that subjects exposed to CT have higher levels of insulin and c-peptide when compared to subjects exposed to none or to severe SLEs only, and that the highest levels are evident in subjects exposed to both CT and severe SLEs.

## Discussion

This is the first study that explored the relationship between exposure to severe stress in childhood and/or adulthood and metabolic dysfunction. Our main finding is that exposure to CT and to both CT and severe SLEs is associated with higher levels of c-peptide and insulin. Our results, even if preliminary, suggest that exposure to CT, acting as a chronic stressor, may increase the levels of insulin and c-peptide, with a further additional effect given by SLEs.

The finding that exposure to stress early in life, as is the case of CT, may make individuals more vulnerable to developing obesity in adulthood and metabolic dysfunctions such as insulin-resistance and diabetes is in line with previous evidence, and further strengthen them (1, 23, 24). The higher levels of insulin we found in subjects exposed to only CT and to both CT and SLEs seem to suggest that CT may act as a “first hit” with the appearance of insulin-resistance, which later can, in turn, bring a rise in insulin levels. The occurrence of SLEs later in life could represent a “second hit,” which would lead to an even more dysfunctional metabolic-response. Since insulin exerts an inhibitory activity on HPA axis (8), such increased levels could represent a compensatory, albeit dysfunctional, attempt to inhibit HPA activity. The c-peptide is a biologically active peptide stored with insulin in the secretory vesicles of the pancreatic β-cells and released by increases in extracellular glucose concentration. While c-peptide does not directly alter glucose metabolism (25), c-peptide and insulin-initiated signaling cascades interact with each other, and this interaction is important for the functioning of both peptides, particularly in erythrocytes (26). Interestingly, the c-peptide plays a role in the tuning of insulin signaling and in endothelial physiology, which could account for the microvascular dysfunction observed in diabetes (27). To date, there is no evidence of altered c-peptide levels in adults exposed to either CT or to SLEs, so our results advance this knowledge about c-peptide. However, data from a prenatal stress animal model (PNS) have found higher levels of c-peptide in adult animals exposed to prenatal stress, although in the absence of alterations in caloric intake, body weight gain or fat mass (28). Intriguingly, our findings are partially in line with these preclinical data (28).

Our finding that levels of insulin and c-peptide are highest in subjects exposed to both CT and SLEs are also partially in line with previous evidence indicating an association between cumulative total stress during lifespan and metabolic dysfunction, specifically with the increase of insulin levels (29). Thus, these findings suggest that a cumulative and repeated stress can lead to an increase of insulin and c-peptide levels, and raise the risk of insulin-resistance, diabetes, and obesity.

The strengths of this study include the evaluation of individuals with no past or current depression, which makes the possibility of a mediation effect between CT and insulin unlikely and the use of standardized instruments to identify childhood trauma, since CECA-Q has good reliability and validity both in general (19) and clinical populations (30, 31). However, since our focus was on CT, we did not explore the possible contribution of emotional abuse, such as parental neglect and antipathy, on glucose metabolism. However, our findings should be taken as preliminary, as the number of subjects with CT and/or SLEs is small. We did not explore the full range of variables known to affect glucose metabolism, as diet regime or daily physical activity, and the presence/absence of diabetes was not confirmed by an oral glucose tolerance test (OGTT). As such, our findings should be replicated in larger samples, specifically evaluated for the presence of diabetes, as well as for both childhood and adult stressors. Also, due to the exploratory nature of the study, we did not apply a correction for multiple comparisons. We believe that even with these limitations, these preliminary findings provide evidence that not only exposure to stress in childhood increases the risk of metabolic dysfunction in adulthood, but also that the additional exposure to severe stress in adulthood may make the individuals even more vulnerable to developing metabolic dysfunctions (1). Based on our preliminary findings, it would be important to investigate the presence of both childhood and adulthood adversity through an accurate and meticulous anamnesis, even when the individual does not present any psychological symptoms. In individuals with a history of adversity, clinicians can implement preventative interventions, such as metabolic investigations for the early detection of insulin resistance, healthy lifestyle education programs, and trauma-specific psychological interventions to limit potential detrimental effects of stress on both mental and physical health.

## Data Availability Statement

The datasets presented in this article are not readily available because the Ethic Committee of the Verona University Hospital did not allow to share datasets. Requests to access the datasets should be directed to Sarah Tosato, sarah.tosato@univr.it.

## Ethics Statement

The studies involving human participants were reviewed and approved by the Ethic Committee of the Verona University Hospital (N. 1995, 31/08/2011; N. 1816, 29/05/2013). The patients/participants provided their written informed consent to participate in this study.

## Author Contributions

STos and AC conceived and designed the study. STos coordinated data collection, interpreted data, and wrote the manuscript. CB performed the statistical analyses and contributed in the interpretation of data. NC, NL, and SP performed the biological analyses. NL and GT contributed in data collection. STom contributed in data collection and in the interpretation of data, and revised the draft critically, together with AC and PD. MR contributed in the interpretation of data. All authors read and approved the final manuscript.

## Conflict of Interest

The authors declare that the research was conducted in the absence of any commercial or financial relationships that could be construed as a potential conflict of interest.
